# Hydrolysis of Whey Protein-Dextran Glycates Made Using the Maillard Reaction

**DOI:** 10.3390/foods8120686

**Published:** 2019-12-15

**Authors:** Na Li, Mark R. Etzel

**Affiliations:** Department of Food Science, University of Wisconsin, 1605 Linden Drive, Madison, WI 53706, USA; nli45@wisc.edu

**Keywords:** conjugation, dairy, carbohydrates

## Abstract

Protein-polysaccharide glycates are food ingredients that use the Maillard reaction to form a Schiff base linkage between the carbonyl of a polysaccharide and the free amino moiety of a protein. Glycates are excellent emulsification, foaming, and gelling agents in foods and improve protein solubility and heat stability. The present work examined if glycates dissociate by hydrolysis, returning to free un-glycated protein and dextran due to the reversibility of the Schiff base linkage. Hydrolysis of glycates made from whey protein isolate and dextran was measured versus time and temperature, allowing determination of the rate constants and equilibrium constants for glycate hydrolysis. Glycates underwent hydrolysis when placed into aqueous solutions at common food processing temperatures. For example, during hot food storage (60 °C), equilibrium fractional hydrolysis was 44%, whereas at ambient temperature (22 °C), it was 8%. The present work aims to increase the successful use of glycates in new foods by knowing what foods and conditions avoid glycate hydrolysis.

## 1. Introduction

The functional properties of proteins in foods such as protein solubility, heat stability, emulsification, foaming, and gelation can be greatly improved by glycation of the proteins by polysaccharides via the Maillard reaction [1,2,3,4,5,6,7,8]. Protein-polysaccharide glycates combine the excellent emulsification properties of proteins with the stabilizing effects of polysaccharides [4]. Rare and threatened sources of emulsifying agents such as gum Arabic might be replaced by protein-polysaccharide glycates [7]. Glycation of proteins as food ingredients may also help people who suffer from food protein allergies by lowering IgE-binding capacity [9].

In protein glycation, a reversible Schiff-base linkage is created between the free amino moiety in the protein and the carbonyl moiety in the polysaccharide via the first step in the Maillard reaction [8,10,11,12,13,14]. The Schiff-base linkage is created either via the dry-heating method or the wet-heating method. In the dry-heating method [7,13,15,16], an aqueous mixture of the protein and polysaccharide is first dried and then heated for a specific time (2 h to 9 d) at a known temperature (60 to 130 °C) and relative humidity (60% to 80%). In the wet-heating method [10,11,17,18,19], the aqueous solution is heated for a certain time (2 h to 2 d) at a set temperature (60 to 95 °C). Past work has centered on the food properties of the glycate in the reaction mixture after heating, but the possibility that the glycates might fall apart in foods by hydrolysis has received little attention.

Hydrolysis is a natural phenomenon resulting from the reversibility of the Schiff-base linkage [20]. Incubation of the protein-polysaccharide glycate in aqueous solution should cause cleavage of the glycate and release of un-glycated protein. In previous studies, glycates made by linkage of whey protein isolate (WPI) and dextran underwent hydrolysis when incubated in water or in aqueous solutions of either dextran or WPI at 62 °C [21]. The time course of the hydrolysis reaction was observed by the measurement of the decrease in the concentration of glycate and the increase in the concentration of un-glycated protein over time.

The purpose of the present study was to more fully explore the time course and equilibrium yield of glycate hydrolysis. Hydrolysis of glycates made from WPI and dextran was examined versus time and temperature. This allowed determination of the rate constants and equilibrium constants for glycate hydrolysis versus temperature. From these constants, the hydrolysis reaction half-life and equilibrium fractional hydrolysis of the glycate were calculated. It was not the purpose of the present work to explore the sequence of elementary chemical reactions that make up the glycate hydrolysis pathway by using the tools of chemical kinetics. Rather, the present work was aimed at measuring the time course and equilibrium yield of the glycate hydrolysis reaction and extracting apparent rate constants using a simple mathematical model and a curve-fitting procedure.

It is important to study the hydrolysis of glycated proteins because hydrolysis can destroy the improved functional properties that glycation was meant to create. The rate and extent of hydrolysis impact the shelf life of foods containing glycated proteins. Wet foods that have high water activity (>0.90), such as beverages, emulsions, gels, and foams, are particularly susceptible to glycate hydrolysis. Furthermore, during glycate formation using the wet-heating method, glycate hydrolysis competes with glycate formation. Developing methods to limit glycate hydrolysis will also assist glycate formation. Therefore, further understanding of glycate hydrolysis is critical to increased understanding of the glycate formation reaction and to increased stability of the glycate in foods. Knowing what foods and conditions avoid glycate hydrolysis will increase the successful use of glycates in new foods.

## 2. Materials and Methods

Whey protein isolate (WPI) was from Davisco Foods International (Le Sueur, MN, USA). WPI contained 92.7% protein, 2.0% ash, 5.0% moisture, 0.0% lactose, and 0.3% lipids. Dextran T10 was from Pharmacosmos Company (Holbaek, Denmark) and had an average molecular mass of 5.2 kDa. Other chemicals were from Fisher Scientific (Pittsburgh, PA, USA). Buffers were prepared at 22 °C.

### 2.1. Synthesis of Glycated Proteins

Glycation was conducted using the dry-heating method, as explained elsewhere (Li, Arunkumar, and Etzel, 2019). Briefly, dextran and WPI were dissolved in 10 mM sodium phosphate buffer (pH 6.5) at a 3:1 mass ratio resulting in a dextran-WPI molar ratio of about 10:1. The liquid reaction mixture was frozen, lyophilized, and ground into a powder using a mortar and pestle. The powder was heated for 16 h at 70 °C in a desiccator that contained saturated potassium chloride solution giving a relative humidity of 80%.

### 2.2. Hydrolysis of Glycated Proteins

For hydrolysis, glycated protein powder was dissolved in 10 mM sodium phosphate buffer at pH 6.5 and divided into 18 tubes each containing 5 mL of the reaction mixture at 5 mg/mL protein (20 mg/mL powder = 5 mg/mL protein + 15 mg/mL dextran). One tube was taken before the start of the hydrolysis reaction (*t* = 0). The remaining tubes were heated for 17 different combinations of time (0 to 192 h) and temperature (60 to 80 °C) in a water bath. Samples were stored at 4 °C before chromatographic analysis.

### 2.3. Chromatographic Analysis

Glycated samples were analyzed by cation exchange chromatography using a 5 mL HiTrap MacroCap SP column from GE healthcare (Marlboro, MA, USA) connected to an ÄKTA Explorer 100 HPLC system (GE Healthcare) [21]. Glycated protein samples were reconstituted in 50 mM sodium lactate, pH 4.0 (Buffer A), and syringe filtered using a 0.22 µm PVDF filter (MilliporeSigma, Burlington, MA, USA). The chromatographic method had 4 steps: (1) equilibrating the column using 5 column volumes (CV) of Buffer A, (2) loading the sample into the column using a 2 mL sample injection loop to bind the glycated and un-glycated proteins to the column, (3) eluting the glycated protein using 12 CV of 40% Buffer B (1 M NaCl in Buffer A), and (4) eluting un-glycated protein using 2.5 CV of 100% Buffer B. See Figures S1 and S2 for example chromatograms. The column was then re-equilibrated using 5 CV of Buffer A and cleaned using 0.1 M NaOH. Glycated protein eluted in the “low salt” peak (40% buffer B) and un-glycated protein eluted in the “high salt” peak (100% buffer B). Unicorn 5.0 software was used to run the chromatographic method and calculate the peak area at 280 nm (*PA_28_*_0_). Protein (µg) ≈ 25 × *PA_28_*_0_, where 25 is the flow rate (5 mL/min) times the path length correction (10 mm/2 mm) for the flow cell.

### 2.4. Kinetic Model of the Hydrolysis Reaction

The hydrolysis of the glycate is the reverse of Schiff base formation in the glycation reaction, as shown in Equation (1) (Li, Arunkumar, and Etzel, 2019):

*PD* + *W* ⇔ *P* + *D* (1)

where *PD* is protein-dextran glycate, *W* is water, *P* is un-glycated protein, and *D* is dextran in Equation (1). Dextran and water were in ten-fold molar excess or more in the present work and essentially constant in concentration. The kinetic expressions are then shown in Equations (2) and (3) [22,23]:

[*PD*] = [*PD*]_eq_ + ([*PD*]_0_ − [*PD*]_eq_) e^−kt^ (2)

[*P*] = [*P*]_eq_ (1 − e^−kt^) (3)

where *k* is the apparent rate constant, and [*PD*] and [*P*] are the concentrations of protein-dextran glycate and un-glycated protein, respectively, at time *t* in Equations (2) and (3). The forward and back rate constants are *k*_1_ and *k*_2_, respectively, and appear in the final model equations as the sum: *k* = *k*_1_ + *k*_2_ [22]. Thus, the rate of approach to equilibrium is determined by the sum of the rate constants (*k*), not by the forward rate constant (*k*_1_). The value of [*PD*] at time zero is [*PD*]_0_. The values of [*PD*] and [*P*] at equilibrium (t → ∞) are [*PD*]*_eq_* and [*P*]*_eq_*, respectively. Measured values of [*PD*] and [*P*] versus time were used to obtain the fitted parameters [*PD*]*_eq_*, [*P*]*_eq_*, and *k*.

The equilibrium constant *K* was calculated from *K =* [*P*]*_eq_*/[*PD*]*_eq_* [24]. The standard free-energy change *∆G°* of the reaction was calculated from the equilibrium constant *K* using the relationship *∆G° = −RT ln*(*K*). The standard enthalpy of formation *∆H°* and the standard molar entropy *∆S°* were calculated from measured values of *K* at three different temperatures using the relationship *∆G° = ∆H°* − *T ∆S°*. The values of *∆G°* and *K* at an unknown temperature were estimated from the values of *∆H°* and *∆S°* [24]. Fractional hydrolysis at equilibrium (*X_eq_*) was calculated using Equation (4) and the stoichiometric relationship [*P*]_eq_ = [*PD*]_0_ − [*PD*]_eq_ in the numerator:

*X_eq_* = [*P*]_eq_/[*PD*]_0_ = *K*/(1 + *K*) (4)

### 2.5. Statistical Analysis

Equations (2) and (3) were fitted to the experimental data of [*PD*] and [*P*] versus time by nonlinear regression using the JMP Pro software, version 11 (SAS Institute, Gary, NC, USA) to obtain the fitted parameter values [*PD*]*_eq_*, [*P*]*_eq_*, and *k*. Results were expressed as mean ± standard error. Pairwise comparisons were made by *t*-test using SAS studio 3.5 (SAS Institute, Gary, NC, USA). The p-value was reported.

## 3. Results

### 3.1. Kinetics of Hydrolysis of the Glycated Protein

**Figure 1:**
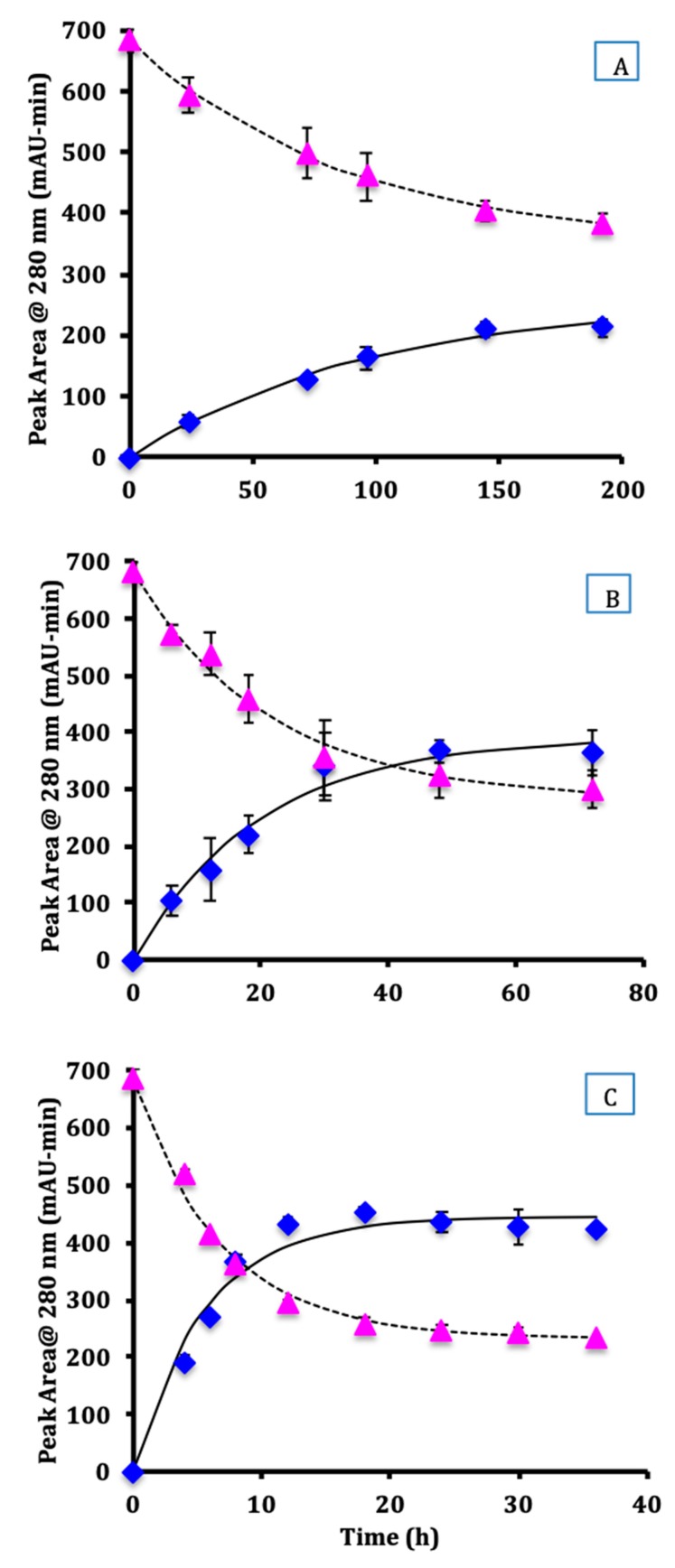
Kinetics of hydrolysis of glycated protein at 60 °C (**A**), 70 °C (**B**), and 80 °C (**C**) for glycated protein (triangles) and un-glycated protein (diamonds). Dotted line is the fit using Equation (2) and solid line using Equation (3). Error bars are standard deviation.

The peak area for glycated and un-glycated protein was measured by chromatographic analysis (Figure 1). At all three temperatures, glycated protein decreased while un-glycated protein increased as reaction time increased, both reaching asymptotic values after a long time.

A first-order reversible reaction model was fit to the data using Equation (2) for glycated protein and Equation (3) for un-glycated protein. The values of the fitted parameters are shown in Table 1.

The apparent rate constants (*k*) measured from the disappearance of glycated protein were not statistically different from the rate constants obtained from the appearance of un-glycated protein at 60 °C, 70 °C, and 80 °C (*p* > 0.05). Thus, the rate of disappearance of glycated proteins was the same as the rate of appearance of un-glycated protein. Increasing the reaction temperature increased the rate of the hydrolysis reaction. The reaction half-life for hydrolysis (*t*_1*/*2_) was 64 h at 60 °C, 14 h at 70 °C, and 4.3 h at 80 °C.

As shown in Table 1, the equilibrium constant (*K*) was calculated from the ratio of the equilibrium concentrations [*P*]*_eq_* and [*PD*]*_eq_*. As the temperature of the hydrolysis reaction increased, the value of *K* increased. Using Equation (4), the equilibrium fractional hydrolysis (*X_eq_*) was 43% at 60 °C, 58% at 70 °C, and 66% at 80 °C.

Rate constants for the forward and reverse reactions of Equation (1), *k*_1_ and *k*_2_, were calculated given that *K* = *k*_1_/*k*_2_ and the apparent rate constant *k* = *k*_1_ + *k*_2_. Both *k*_1_ and *k*_2_ increased with increasing temperature. The Arrhenius activation energy was 154 ± 11 kJ/mol for *k*_1_ and 107 ± 3 kJ/mol for *k*_2_. Higher temperatures favored the forward (hydrolysis) reaction compared to the back reaction.

### 3.2. Thermodynamic Analysis of the Hydrolysis Reaction

**Figure 2:**
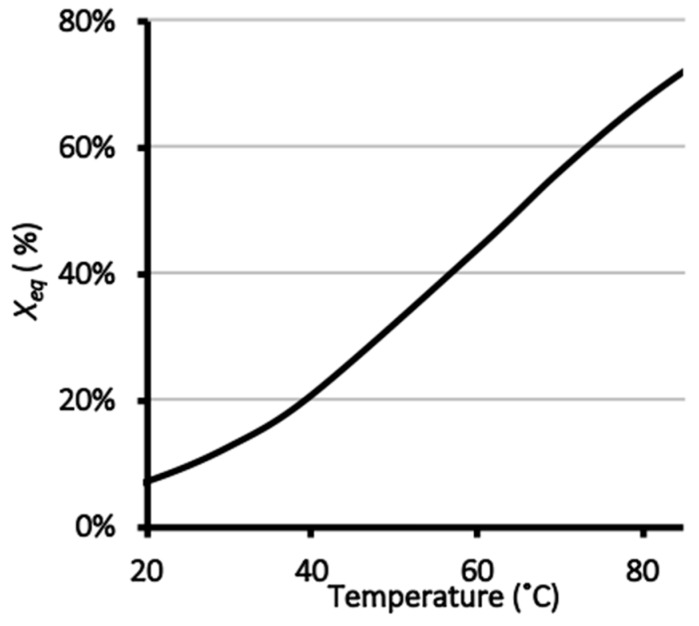
Calculated equilibrium fractional hydrolysis (*X_eq_*) versus temperature.

The values of *K* at each temperature were used to calculate *∆G°* for the hydrolysis reaction. Plotting *∆G°* versus temperature yielded a straight line with intercept *∆H°* = 47 ± 8 kJ/mol and slope *∆S°* = 0.14 ± 0.02 kJ/(mol·°K). From *∆H°* and *∆S°*, the values of *K* and *X_eq_* at any other temperature were calculated (Figure 2). As temperature increased, *X_eq_* increased, reaching 72% at 85 °C. Conversely, *X_eq_* decreased to 8% at 22 °C. Thus, glycated proteins were relatively stable at ambient temperatures and below, whereas most of the glycated protein underwent hydrolysis above 65 °C.

## 4. Discussion

In aqueous solution, glycated proteins fell apart over time and underwent hydrolysis back to un-glycated proteins. Glycates fell apart more quickly and to a greater equilibrium extent as the temperature was increased. Common temperatures used in thermal processing of foods may cause significant glycate hydrolysis.

One solution to the hydrolysis problem is to use a food-grade reducing agent after the formation of the glycate. Glycate formation and hydrolysis are synonymous with Schiff base formation and hydrolysis, a well-understood phenomenon [20,25]. Unstable Schiff bases are reduced to stable secondary amines by sodium cyanoborohydride and sodium borohydride [26]. Sodium cyanoborohydride is highly toxic and not safe for use in foods. Sodium borohydride, a food-grade chemical, is sometimes used to reduce the Schiff base, but it is less effective than sodium cyanoborohydride. Ascorbic acid, another food-grade chemical, can also convert the Schiff base to a stable secondary amine, but it is less effective than sodium borohydride [27,28]. Another advantage of Schiff base reduction is that if done during glycate formation by the wet-heating method, it removes the glycate from the solution. This drives the reaction to completion by shifting the equilibrium towards the glycate, according to Le Chatelier’s principle [23], increasing the yield of the glycate. Low glycate yield is one of the disadvantages of the wet-heating method compared to the dry-heating method [29].

## Figures and Tables

**Table 1:** Parameter values from fitting Equations (2) and (3) to the data of Figure 1.

Temperature (°C)	60	70	80
Glycated protein *k* (h^−1^)	0.0115 ± 0.0007	0.046 ± 0.006	0.15 ± 0.01
Un-glycated protein *k* (h^−1^)	0.010 ± 0.002	0.051 ± 0.007	0.18 ± 0.02
[*PD*]*_eq_* (mAU-min)	345 ± 9	280 ± 19	231 ± 9
[*P*]*_eq_* (mAU-min)	255 ± 18	392 ± 21	447 ± 16
*K*	0.74 ± 0.06	1.4 ± 0.1	1.9 ± 0.1
*k*_1_ (h^−1^)	0.00463 ± 0.00001	0.0283 ± 0.0003	0.107 ±0.003
*k*_2_ (h^−1^)	0.006 ± 0.001	0.020 ± 0.004	0.055 ± 0.008

## Supplementary Materials

The following are available online at https://www.mdpi.com/2304-8158/8/12/686/s1, Figure S1: Chromatogram of sample taken before hydrolysis showing that at 0 h the sample contained mainly glycated protein [PD] and little un-glycated protein [P]. Figure S2: Chromatogram of sample taken at a hydrolysis time of 30 h and temperature of 80 °C showing the disappearance of glycated protein [PD] and appearance of un-glycated protein [P].
