# Supplementary material for: Hydrolysis of Whey Protein-Dextran Glycates Made Using the Maillard Reaction
Source: Foods. 2019 Dec 15;8(12):686. doi: 10.3390/foods8120686 (PMC6963623; doi:10.3390/foods8120686)
Supplement: Supplementary file 1 [file foods-08-00686-s001.pdf]

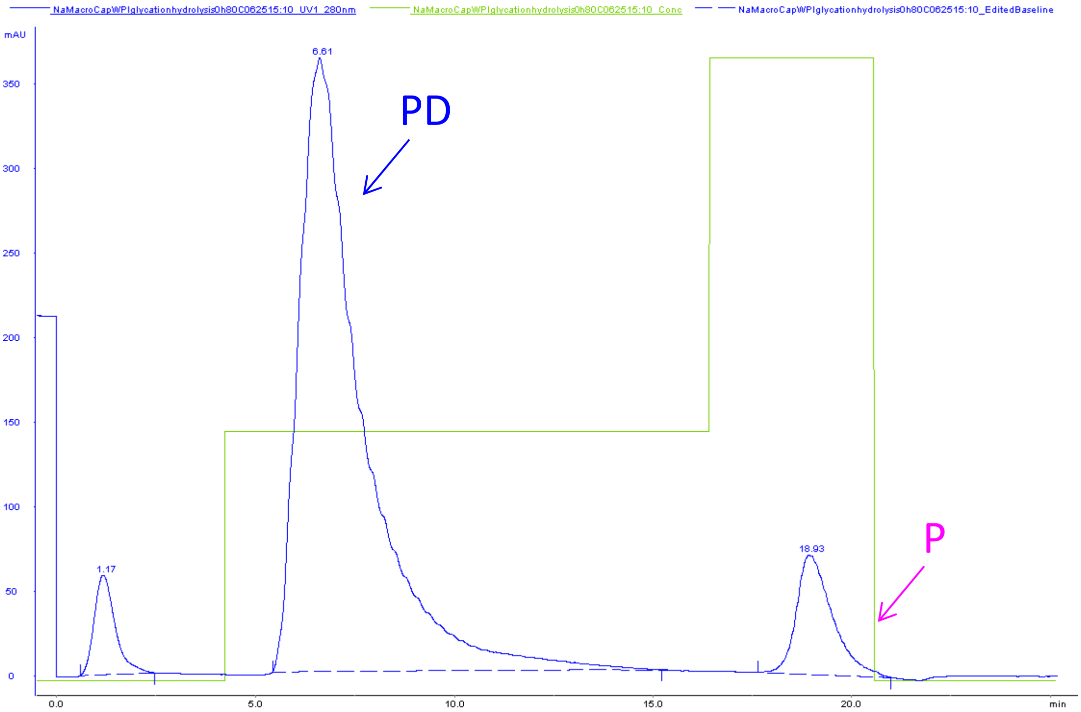

Figure S1. Chromatogram of sample taken before hydrolysis showing that at 0 h the sample contained mainly glycated protein [PD] and little un-glycated protein [P].

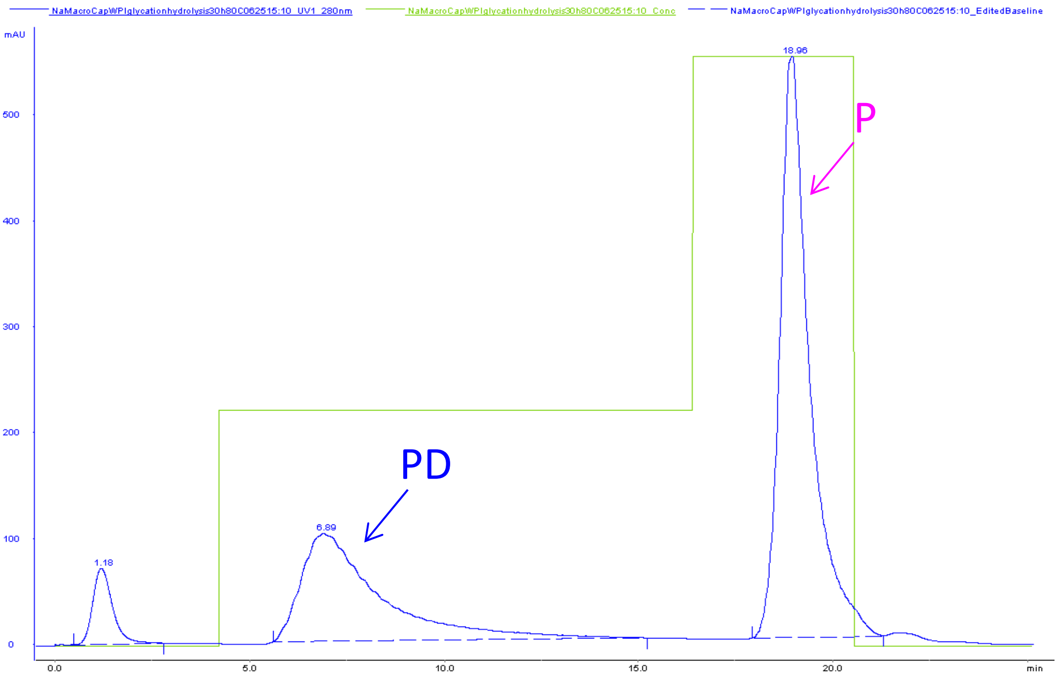

Figure S2. Chromatogram of sample taken at a hydrolysis time of 30 h and temperature of 80 °C showing the disappearance of glycated protein [PD] and appearance of un-glycated protein [P].
